# Programming of Regulatory T Cells In Situ for Nerve Regeneration and Long-Term Patency of Vascular Grafts

**DOI:** 10.34133/2022/9826426

**Published:** 2022-07-19

**Authors:** Yanhong Wang, Fangchao Xue, Yanzhao Li, Lin Lin, Yeqin Wang, Shanlan Zhao, Xingli Zhao, Yong Liu, Ju Tan, Gang Li, Haoran Xiao, Juan Yan, Hao Tian, Min Liu, Qiao Zhang, Zhaojing Ba, Lang He, Wenyan Zhao, Chuhong Zhu, Wen Zeng

**Affiliations:** ^1^Department of Cell Biology, Third Military Army Medical University, Chongqing 400038, China; ^2^Department of Anatomy, National and Regional Engineering Laboratory of Tissue Engineering, State and Local Joint Engineering Laboratory for Vascular Implants, Key Lab for Biomechanics and Tissue Engineering of Chongqing, Third Military Medical University, Chongqing 400038, China; ^3^State Key Laboratory of Trauma, Burn and Combined Injury, Chongqing, China; ^4^Departments of Neurology, Southwest Hospital, Third Military Medical University, Chongqing, China

## Abstract

Rapid integration into the host tissue is critical for long-term patency after small diameter tissue engineering vascular grafts (sdTEVGs) transplantation. Neural recognition may be required for host integration and functionalization of the graft. However, immune rejection and inflammation hinder nerve regeneration of sdTEVGs. Here, a CRISPR/dCas9-nanocarrier was used for targeted programming of regulatory T cells (Treg cells) in situ to promote nerve regeneration of sdTEVGs by preventing excessive inflammation. Treg cells and (C-C chemokine receptor) CCR2+ macrophage recruitment occurred after transplantation. The nanodelivery system upregulated ten eleven translocation (TET2) in Treg cells in vitro. Reprogrammed Treg cells upregulated anti-inflammatory cytokines and decreased the proportion of CCR2+ macrophages. IL-6 concentrations decreased to the levels required for nerve regeneration. Implantation of CRISPR/dCas9 nanodelivery system-modified sdTEVGs in rats resulted in Treg cell editing, control of excessive inflammation, and promoted nerve regeneration. After 3 months, nerve regeneration was similar to that observed in normal blood vessels; good immune homeostasis, consistency of hemodynamics, and matrix regeneration were observed. Neural recognition promotes further integration of the graft into the host, with unobstructed blood vessels without intimal hyperplasia. Our findings provide new insights into vascular implant functionalization by the host.

## 1. Introduction

Small diameter tissue engineering vascular grafts (sdTEVGs; <4 mm) are in high demand for treating cardiovascular diseases or for hemodialysis [1]. However, the association with a high incidence of thrombosis, intimal hyperplasia, and calcification has hindered their clinical application. In particular, the use of sdTEVGs in the size range of 1.5 to 2 mm during coronary artery bypass still poses many challenges [2–6]. Imbalance in vascular homeostasis (i.e., aneurysm, intimal hyperplasia, and calcification) remains a challenge following the endothelialization of sdTEVGs [7]. Thus, the long-term, steady-state formation of sdTEVGs requires further investigation.

In an autogenous environment, nerves and blood vessels influence each other. Practically all blood vessels of the human body, except capillaries, are innervated by sympathetic vasoconstrictor fibers [8–10]. Nerves can mediate the proliferation and contractile activity of vascular smooth muscle cells through a variety of transmitters and regulate their normal phenotype. We have previously demonstrated that neurotrophic factors promote endothelialization and effectively improve the patency rate of sdTEVGs [11, 12]. An sdTEVG implantation procedure will inevitably lead to peripheral nerve injury; thus, the effective induction of nerve fiber regeneration is a key problem that needs to be addressed to promote long-term patency and homeostasis of sdTEVGs in vivo.

As acellular vascular grafts retain the structure and composition of native blood vessels following decellularization, they have favorable mechanical properties and are suitable for integrating sdTEVGs [13]. Foreign implants may induce the recruitment of various types of immune cells, such as T cells and monocytes/macrophages, 30 days postimplantation. The infiltration of macrophages, especially CCR2+ macrophages associated with monocyte recruitment, can cause an increase in the levels of multiple inflammatory factors, leading to excessive immune rejection, thrombosis, rapid degradation of the vascular graft, and inhibition of nerve reconstruction [14, 15]. However, inflammation-mediated remolding of the graft can also occur during this process [14–16]. Regulatory T (Treg) cells are immunosuppressive T cells that can counteract excessive inflammation, maintain immune homeostasis [17], modulate the proliferation and differentiation of tissue-resident stem cells after injury, and play important roles in organ transplantation [18–20]. Ten eleven translocation (TET) proteins and DNA modification exert a pervasive influence on immune cell development and function. The TET2-catalyzed conversion of 5-methylcytosine to 5-hydroxymethylcytosine in Foxp3 establishes a Treg cell-specific hypomethylation pattern. TET2 deficiency leads to Foxp3 hypermethylation, causing impaired Treg cell function in regulating excessive inflammation [21, 22]. Thus, it is important to determine whether stable TET2 and Foxp3 expression in Treg cells can regulate excessive inflammation in the local area and induce peripheral nerve fiber regeneration of sdTEVGs.

Here, we constructed a CRISPR/dCas9 nanodelivery system for the targeted programming of Treg cells to promote nerve regeneration of sdTEVGs that transplanted in rats by regulating excessive inflammation in situ. This system targeted the Treg cell surface marker CD25 to promote TET2 expression and maintain immune homeostasis. We demonstrated that the targeted delivery system specifically regulated immune homeostasis by stabilizing Foxp3 expression in Treg cells in vivo, repolarizing macrophages, inhibiting excessive inflammatory response, and regulating interleukin- (IL-) 6 level secreted by macrophages to achieve nerve reconstruction and long-term patency of sdTEVGs. This advanced implant system with immunomodulatory function is also suitable for use in other fields as it provides universal guidance for circumventing the wide range of adverse effects induced in response to immune rejection and promotes the integration of the graft into the host tissue.

## 2. Results and Discussion

### 2.1. Tregs and CCR2+ Macrophages Infiltrated after sdTEVG Transplantation

**Figure 1:**
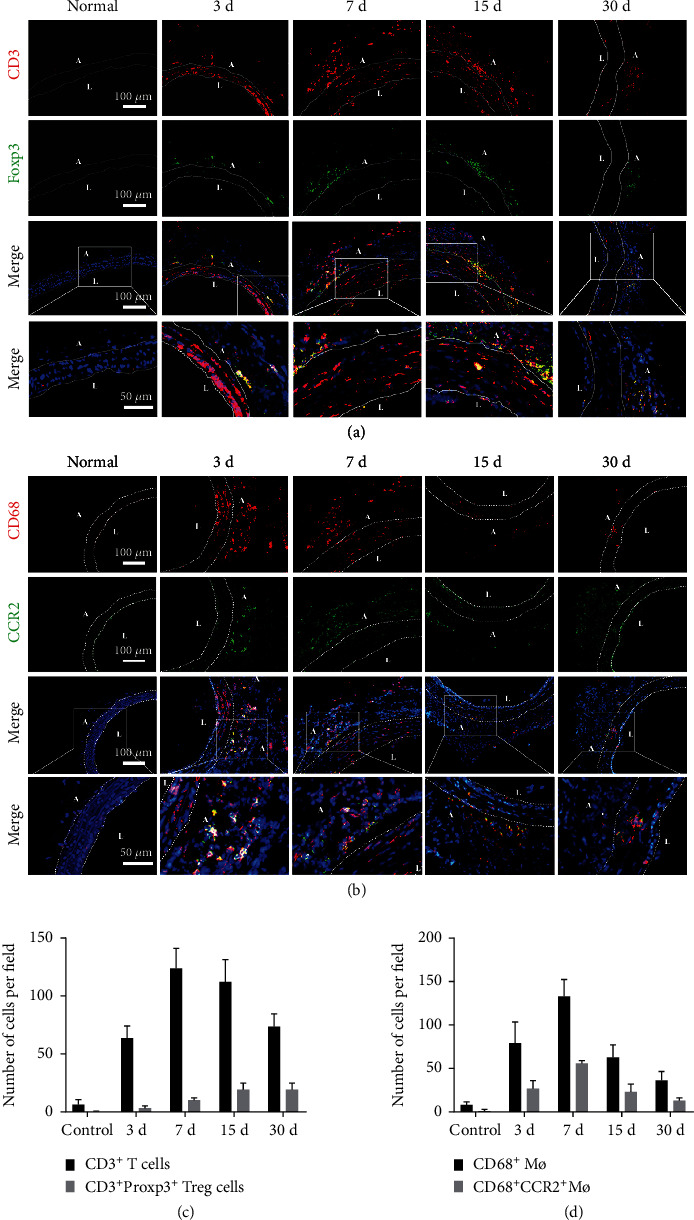
Stability of immune cell infiltration after sdTEVG transplantation. Immunofluorescence staining to check the infiltration of immune cells in transplanted sdTEVGs. (a) T cells (CD3 and DAPI) and Treg cells (CD3, Foxp3, and DAPI). (b) Macrophages (CD68, CCR2, and DAPI). The dot lines mean dividing lines between the vascular adventitia, media, and intima. A and L means adventitia and lumen of sdTEVGs. (c, d) Mean number of immune cells in each visual field within 30 days of vascular transplantation. *n* = 5, data are presented as means ± SD.

During inflammation, M1 and M2 macrophages may promote the healing of injured tissues by phagocytizing cell debris, resulting in the release of specific cytokines and growth factors [12, 23, 24]. Recently, it has been revealed that C-C chemokine receptor 2- (CCR2-) macrophages might be involved in wound healing and regeneration [25]. Molecular and cellular events that occur during the early steps of nerve regeneration include axonal fragmentation and phagocytosis by invading macrophages. To detect immune cell infiltration, immunofluorescence was performed on frozen sdTEVG sections at different time points after transplantation into Sprague-Dawley (SD) rats (Figures 1(a) and 1(b)). Three days after transplantation, the absolute number of T cells began to increase, peaking on day 7, and this trend was maintained for the next 15 days. The numbers of CD3 + Foxp3 + Treg cells peaked on day 30 (Figure 1(c)). Meanwhile, the trend in the change in macrophage (Mø) counts was similar to that observed for Treg cells. CD68 + Mø and CD68 + /CCR2 + Mø counts increased over time and peaked on day 7 (Figures 1(b) and 1(d)). Treg cells and CCR2+ macrophages were recruited after transplantation, and both cells persisted for a long duration more than 30 days. Subsequently, the macrophage counts began to decline between days 7 and 30, indicating that both were synchronously involved in early inflammation around the graft.

### 2.2. The CRISPR/dCas9 Nanodelivery System Activated Treg Cells and Inhibited Excessive Inflammation

**Figure 2:**
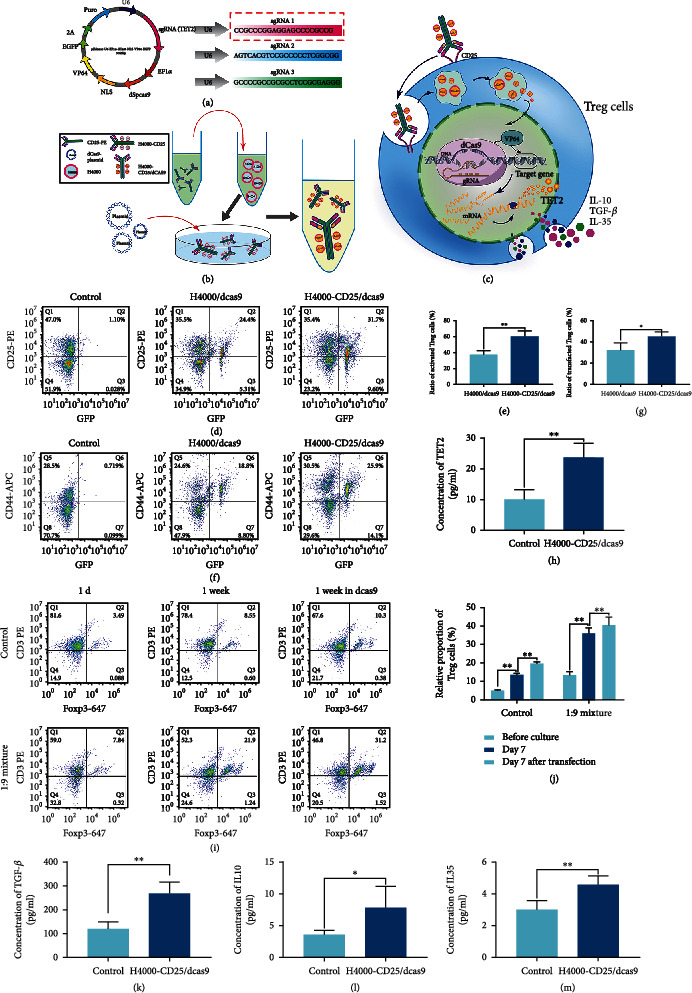
CRISPR/dCas9 nanodelivery system used to activate Treg cells and inhibit excessive inflammation in vitro. (a) Design of the plasmid showing all the minimal modules, including the three versions of the sgRNA. The red box shows the sequence used. (b) Schematic diagram of the gene delivery system. CD25-PE covalently binds cationic polymer H4000 as the targeted transfection vector. After adding dcas9-plasmids to the H4000-CD25 suspension dropwise, H4000-CD25/dCas9 nanoparticles packaging dCas9 plasmids were obtained. (c) A simple schematic illustration of the active Treg cell targeting mechanism of the nanogene editing system. (d, g) The transfection efficiency of Treg cells was confirmed using flow cytometry, control group means Treg cells product with no transfection; *n* = 6, data were analyzed using unpaired Student's *t*-test. (e, f) The percentage of activated Treg cells after transfection was characterized using flow cytometry, *n* = 6, one- way ANOVA followed by Tukey test. (h) ELISA was used to detect the TET2 protein content before and after Treg cell transfection. From top to bottom, *n* = 6, unpaired Student's t-test. (i, j) Mixed lymphocyte reaction. T cells from rat spleens were transfected and cultured for 7 days. Furthermore, natural T cells and different transfection treatment Treg cells were cocultured at a 1 : 9 ratio for 7 days. The ratio of T cells to Treg cells was detected using flow cytometry to reveal the relative proliferation rate of Treg cells, *n* = 6, two- way ANOVA followed by Tukey test. (k–m) The level of anti-inflammatory cytokines in Treg cells before and after transfection with the nanodelivery system. From left to right: TGF-*β*-ELISA, IL-10 ELISA, and IL-35-ELISA. *n* = 6, unpaired Student's *t*-test. ^∗^*P* < 0.05, ^∗∗^*P* < 0.01, n.s.: no significance. Data are presented as means ± SD. Above control group means normal group with no transfection.

Previous experiments have shown that the expression of TET2 in Treg cells can be increased in response to treatment with the TET2 agonist, vitamin C, and decreased in response to treatment with the inhibitor SC-1 (Supplementary Figure 1). To enhance TET2 expression in Treg cells in vivo and in vitro, a nonviral plasmid DNA delivery system containing an enhanced TET2 gene promoter named as dcas9 plasmid, cationic polymers H4000 as transfection vector, and targeting peptides was designed (Figures 2(a)–2(c)). After transfecting the cells with the plasmid, the presence of the reporter (i.e., green fluorescent protein (GFP)) indicated successful transfection in vivo (Supplementary Figure 2). Flow cytometry revealed that the transfection (Foxp3 and GFP positive Treg cells) and activation efficiencies (CD44 + GFP + Treg cells) were significantly increased after modification of the CRISPR/dCas9 nanodelivery system with the CD25 antibody (Figures 2(d)–2(g); Supplementary Figure 2). The increase in intracellular TET2 protein levels was indicative of improved gene delivery (Figure 2(h)). These results indicated the superior targeting of the CD25 antibody-modified NPs (H4000-CD25/dCas9) to that of the unmodified NPs (H4000/dCas9). The CD25 antibody enhanced the targeted editing efficiency of the gene delivery system. The mixed lymphocyte reaction is a commonly used method in immune modulation studies. We tried to determine the contribution of Treg cells in counteracting the excessive immune response. The results showed that the proportion of Treg cells in T cells increased significantly, especially in cells transfected with H4000-CD25/dCas9 after coculturing T cells and Treg cells at a ratio of 1 : 9 for 7 days (Figures 2(i) and 2(j)). Moreover, the levels of inflammatory inhibitory factors, including IL-10, IL-35, and TGF-*β*, secreted by Treg cells were also elevated following transfection with H4000-CD25/dCas9 (Figures 2(k)–2(m)). These data suggested that Treg cells inhibited the proliferation of T-lymphocytes by secreting inhibitory cytokines, and that the immunosuppressive function of active-Treg cells was enhanced following transfection with the H4000-CD25/dCas9 gene nanodelivery system via TET2 expression upregulation. This confirmed the potential ability of activated Treg cells to inhibit transplant-induced T cell-mediated immune rejection and excessive inflammation.

### 2.3. Decreased CCR2+ Macrophage Counts in Response to Activated Treg Cells Promoted Nerve Regeneration

**Figure 3:**
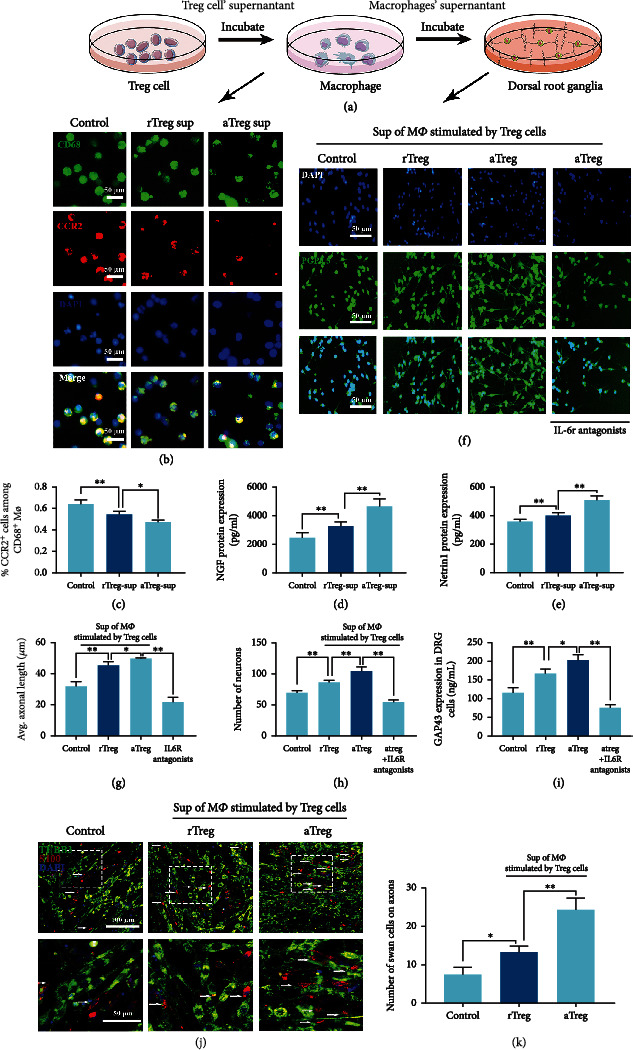
Activated Treg cells promoted nerve regeneration by regulating CCR2+ macrophages. (a) Cartoon photo of experiment process in cell supernatants obtaining and function evaluating of nerve regeneration. (b) Rat macrophages incubated in Treg cell supernatants were visualized by immunofluorescence staining. rTreg sup: supernatant of regular Treg cells; aTreg sup: supernatant of Treg cells activated by H4000-CD25/dCas9 nanodelivery system. Green: CD68; red: CCR2; blue: DAPI. (c) Percentage of CCR2+ macrophages among CD68 macrophages. *n* = 6, one-way ANOVA followed by Tukey test. (d, e) The level of nerve growth-related factors (NGF) and Netrin-1 secreted by macrophages stimulated by the Treg cell supernatant for 2 days. *N* = 6, one- way ANOVA followed by Tukey test. (f) Immunofluorescence staining: the rat dorsal root ganglion incubated with supernatant of macrophages separately stimulated by rTreg and aTreg cells and a group of macrophage supernatant added with IL-6 antagonist. Blue: DAPI and green: PGP9.5. (g, h) Axon length and cell survival of DRG after coculturing with macrophage supernatants from each group, *n* = 6, one-way ANOVA followed by Tukey test. (i) GAP43 expression in DRG cells after coculturing with macrophage supernatant from each group. *n* = 6, one- way ANOVA followed by Tukey test. (j) Schwann cells and DRG cells were cocultured and then incubated with the supernatants of macrophages stimulated by rTreg and aTreg cells. (k) Number of Schwann cells grown on axons of DRG cells in each visual field, *n* = 12, one- way ANOVA followed by Tukey test. ^∗^*P* < 0.05, ^∗∗^*P* < 0.01, n.s.: no significance. Data are presented as means ± SD.

To verify that edited Treg cells can regulate the inflammatory environment, we incubated rat macrophages for 2 days with the supernatant of Treg cells transfected with H4000-CD25/dCas9 (active Treg cells, aTreg-sup), Treg cells with no transfection (rest Treg cells, rTreg-sup), or with RPMI medium (control), and then, new macrophages supernatant in different groups were extracted out to detect the function on nerve regeneration and factor mechanism (Figure 3(a)). The results showed that the proportion of CCR2+ macrophages, which was associated with monocyte recruitment and inflammation, decreased after treatment with aTreg-sup (Figures 3(b) and 3(c)). In addition to phagocytosing degenerated nerve cell fragments, CCR2- macrophages can promote nerve regeneration through paracrine stimulation [26, 27]. We found that the levels of nerve growth-related factors NGF and Netrin-1 in the macrophage supernatant were significantly increased after stimulation with both rTreg-sup and aTreg-sup, especially the latter (Figures 3(d) and 3(e)). Although the role of Netrin-1 and NGF secreted by macrophages in nerve regeneration has been demonstrated [14, 15], IL-6, a typical inflammatory factor derived from macrophages and other immune cells, has been found to be significantly involved in peripheral nerve regeneration [28, 29]. Thus, to investigate the effect and mechanism of CCR2- macrophages on nerve regeneration, we incubated dorsal root ganglion (DRG) neurons with the three groups of macrophage supernatants above and added an IL-6 receptor antagonist group (Figure 3(f)). Compared with that in the control and rTreg-sup groups, the CCR2- macrophage supernatant group exhibited promotion of axon regeneration, and after treatment with the IL-6 receptor antagonist, axon regeneration was significantly reduced (Figures 3(g) and 3(h)). ELISA for neuronal axon surface growth-related protein 43 (GAP43) also showed the same trend (Figure 3(i)). Interestingly, we found that as the gradient of IL-6 changed, the length and number of axons showed a parabolic trend that was initially enhanced and then inhibited, and the optimal IL-6 concentration was 50–100 ng mL-1 (Supplementary Figure 3). Therefore, we speculated that the excessive secretion of IL-6 by CCR2+ macrophages inhibited DRG neuron axon regeneration. With the decrease in the level of IL-6, nerve growth gradually entered the most suitable phase, and continued decrease of IL-6 weakened the trend of nerve growth. Thus, IL-6 may have played a more decisive role in this process than NGF or Netrin-1. Regulating the proportion of CCR2 macrophage subsets by editing Treg cells may increase the local concentrations of IL-6 in the optimal range for nerve regeneration. Furthermore, the regeneration of nerve axons requires the support of Schwann cells, which phagocytose axonal debris and secrete beneficial growth factors, such as pleiotrophin and glial cell-derived neurotrophic factor [30]. To explore the influence of the inflammatory microenvironment on Schwann cells, we cocultured primary rat DRGs and Schwann cells to study their combination and nerve regeneration.

The expression of S100 in the different groups showed that the supernatant of macrophages stimulated by Treg cells matched the combination of cells, especially as Treg cells could significantly promote the growth of Schwann cells on axons (Figures 3(j) and 3(k)).

### 2.4. Design and Properties of sdTEVGs Modified with H4000-CD25/dCas9 Nanodelivery System

**Figure 4:**
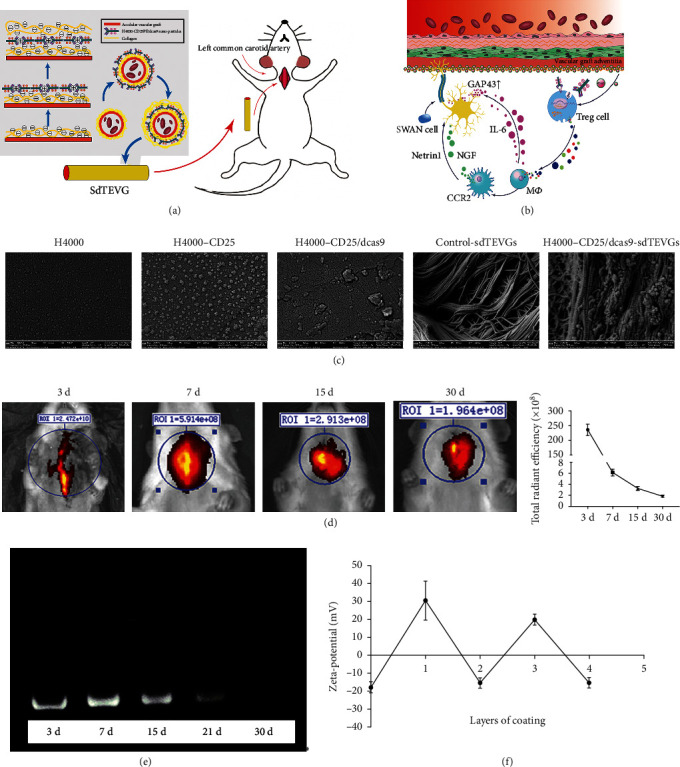
Design and properties of sdTEVGs modified with H4000-CD25/dCas9 nanodelivery system. (a) Schematic diagram of the nanodelivery system targeting Treg cells to control inflammation and promote nerve regeneration on sdTEVGs. (b) Construction of the long-term sustained-release vascular adventitia engineering design using an electrostatic layer-by-layer self-assembled nanodelivery system. (c) SEM showed the process of H4000 nanoparticles modification on sdTEVGs adventitia after dCas9 delivery. (d) The delivery system loaded with DIR dye replaced the left common carotid artery of rats. The fluorescence attenuation within 30 days was measured. *n* = 5. (e) Plasmids continuously released from sdTEVGs in agarose gel for 30 days, detected using qPCR. (f) The surface zeta potential of electrostatic self-assembly of an engineered blood vessel. Layers 1 and 3 represent the H4000-CD25/dCas9 layer, and layers 0, 2, and 4 represent the hyaluronic acid (HA) layer. *n* = 6, data are presented as means ± SD.

Based on these results, the protonated H4000-CD25/dCas9 delivery system and collagen with many electrons were used to construct a long-term sustained-release engineering vessel using an electrostatic layer-by-layer self-assembly method (Figures 4(a) and 4(b)). The successful modification on the adventitia of the decellularized vascular matrix was confirmed by surface zeta potential analysis and scanning electron microscopy. After encapsulating the plasmid, the H4000-CD25/dCas9 system appeared in clusters, and the sdTEVGs modified with H4000-CD25/dCas9 showed that many nanoparticles were adsorbed onto the adventitia surface (Figures 4(c) and 4(f)). To evaluate the sustained-release efficiency and maintenance time, we added fluorescent molecules (Cy3 or Dir) to H4000 (Supplementary Figure 4), and then, sdTEVGs modified with H4000-Cy3 or DiR were placed in agarose gel or implanted in rats. After observation for a period, the modified sdTEVGs showed strong fluorescence at days 7 and 15, which was maintained until day 30 both in vitro and in vivo (Figure 4(d); Supplementary Figure 4). With respect to the in vitro characterization of the sustained-release of the CRISPR/dCas9 plasmid, qPCR revealed that the plasmid was continuously released for over 21 days (Figure 4(e)). These data suggest that the system could maintain the sustained release for nearly 1 month in vivo. Thus, it provided sufficient time for nerve fibers to grow into sdTEVGs.

### 2.5. The Modified sdTEVGs Targeted Programming of Treg Cells In Vivo and Decreased the Proportion of Infiltrative CCR2+ Macrophages

**Figure 5:**
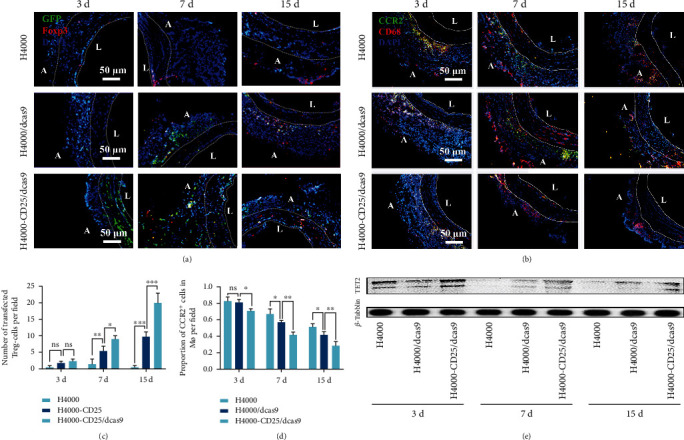
The H4000-CD25/dCas9 delivery system for sdTEVGs targeted Treg cells in vivo and decreased the proportion of CCR2+ macrophages. (a) The H4000-CD25/dCas9 delivery system was transfected into Treg cells infiltrated on sdTEVGs, green: GFP; red: Foxp3; blue: DAPI; *n* = 5. (b) Immunofluorescence staining of sdTEVGs showed the decreased adventitia infiltration of CCR2+ macrophages in the H4000-CD25/dCas9 group, green: CCR2, red: CD68, blue: DAPI; *n* = 5. The dot lines mean dividing lines between the vascular adventitia, media, and intima. A and L means adventitia and lumen of sdTEVGs. (c) Transfection efficiency of the H4000-CD25/dCas9 delivery system into Treg cells. *n* = 5, two- way ANOVA followed by Tukey test. (d) The number of CCR2+ macrophages in the adventitia of sdTEVGs. *n* = 5, two- way ANOVA followed by Tukey test. (e) Western blot showed the TET2 protein in sdTEVGs in three groups at different time points. ^∗^*P* < 0.05, ^∗∗^*P* < 0.01, ^∗∗∗^*P* < 0.001, n.s.: no significance. Data are presented as means ± SD.

After transplantation of H4000-CD25/dCas9-modified sdTEVGs into the common carotid artery of SD rats using confocal immunofluorescence, we observed that the number of GFP+ and Foxp3+ Treg cells in the H4000-CD25/dCas9 group was distinctly larger than that in the control and H4000/dCas9 groups over time. This confirmed the successful targeting and transfection of the H4000-CD25/dCas9 system in Treg cells in vivo (Figures 5(a) and 5(c)). After the transfected Treg cells infiltrated in grafted vessels, dCas9 significantly promoted their TET2 expression on days 3, 7, and 15 (Figure 5(e)). Thus, immune homeostasis could be regulated by Treg cells with stabilized Foxp3 expression in vivo. Furthermore, we examined macrophage infiltration in sdTEVGs and found that the ratio of CCR2+ macrophages decreased over time, especially in the H4000-CD25/dCas9 group (Figures 5(b) and 5(d)). These results showed that the H4000-CD25/dCas9 delivery system could shift the macrophage phenotype toward the CCR2- phenotype by inducing stable Foxp3 expression in Treg cells in vivo. Thus, our system provided an appropriate microenvironment for nerve regeneration.

### 2.6. The H4000-CD25/dCas9 Delivery System Induced Nerve Regeneration and Remodeling of sdTEVGs

**Figure 6:**
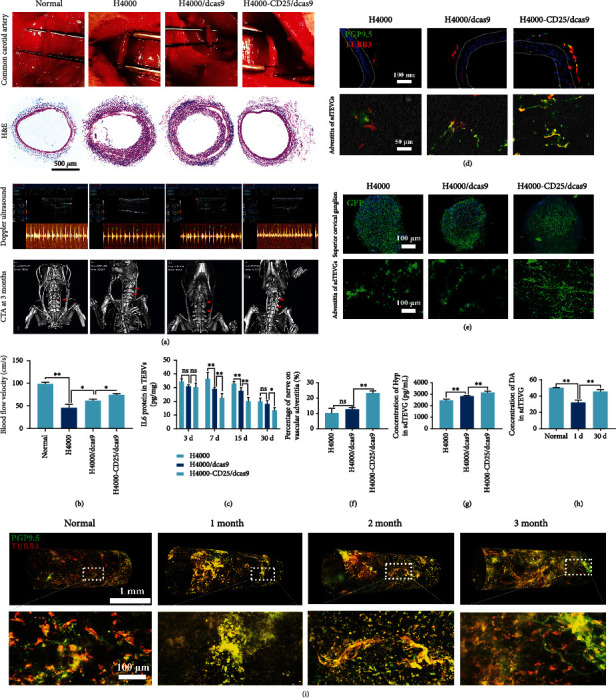
The H4000-CD25/dCas9 delivery system promotes nerve regeneration and matrix remodeling of sdTEVGs in rats. (a) From top to bottom: the engineered blood vessels were used to replace the left common carotid artery of rats; 30 days after transplantation, the frozen sections of sdTEVGs were stained with hematoxylin and eosin (H&E); ultrasonic Doppler examination of left sdTEVGs in rats; 90 days after transplantation, computed tomography angiography (CTA) was performed in the rat neck. Arrows pointed to sdTEVGs. (b) Thirty days after transplantation, the blood flow velocity in sdTEVGs in rats was statistically analyzed. *n* = 5, one-way ANOVA followed by Tukey test. (c) On day 30 of transplantation, ELISA tested the IL-6 contents of sdTEVGs in three groups at various time points. *n* = 5, two- way ANOVA followed by Tukey test. (d) Nerve immunofluorescence staining of frozen sections of sdTEVGs on day 30 after transplantation, green: PGP9.5, red: TUBB3. (e) On day 30, after the virus tracer was injected into the stellate ganglion of rats, the stellate ganglion (upper) and the adventitia nerve of the sdTEVGs expressed green fluorescent protein (below). *n* = 5, one- way ANOVA followed by Tukey test. (f) Statistics of nerve coverage on sdTEVGs adventitia. *n* = 5, one- way ANOVA followed by Tukey test. (g, h) Hydroxyproline (Hyp) kits and DA ELISA kits tested the collagen and dopamine content of sdTEVGs. *n* = 5, one- way ANOVA followed by Tukey test. (i) Thirty days after transplantation, whole vascular adventitia was performed with nerve immunofluorescence staining and subsequent three-dimensional reconstruction, green: PGP9.5, red: TUBB3. ^∗^*P* < 0.05, ^∗∗^*P* < 0.01, n.s.: no significance. Data are presented as means ± SD.

SdTEVGs modified with the H4000-CD25/dCas9 nanodelivery system were transplanted into SD rats to detect the effects of the dCas9 sustained-release system on their patency and nerve remodeling (Figure 6(a)). Abundant adventitia tissue was observed by hematoxylin and eosin (H&E) staining at day 30, which might have been caused by the infiltration of inflammatory cells and migration of adventitia vascular progenitor cells. Intravascular stenosis and accumulation of inflammatory cells in the intima could be observed in the groups, except for the H4000-CD25/dCas9 group. Our findings were confirmed using Doppler ultrasound imaging and computed tomographic angiography (CTA) to evaluate blood flow and patency. Compared with that in the control groups, the H4000-CD25/dCas9 group showed the best vascular patency rate (Figure 6(a)). Doppler ultrasound revealed that the H4000-CD25/dCas9 group exhibited stable hemodynamics as the blood flow velocity of sdTEVGs remained stable at 83 ± 6.4 cm s − 1 after 30 days (Figure 6(b)). Furthermore, CTA showed that the H4000-CD25/dCas9-modified sdTEVGs remained unobstructed in the 3 following months.

Furthermore, we explored whether sdTEVGs play a role in maintaining immune homeostasis in the microenvironment and vascular remodeling. The expression of inflammatory factors, such as IL-6, began to decrease on day 7 and was still lower than that in the control group on day 30 (Figure 6(c)), demonstrating that sdTEVGs exhibited a lower level of inflammation. On day 30 of transplantation, the nerve fibers in the adventitia of blood vessels, as demonstrated in the confocal images (Figure 6(d)), showed nerve regeneration in the adventitia. After tracer labeling of the superior cervical ganglion using virus injection, a GFP signal was observed on the sdTEVGs adventitia 60 days after transplantation, suggesting that nerves regrew from the superior cervical ganglion, and that the intensity of the GFP fluorescence signal was significantly increased, especially in the H4000-CD25/dCas9 group (Figures 6(e) and 6(f)). The results indicated that excessive inflammation was regulated by Treg cells and that the IL-6 level was decreased to an appropriate level for nerve regeneration by the H4000-CD25/dCas9 system in vivo.

As a neurotransmitter and an important substance in the maintenance of the structural strength of blood vessels, the content of vascular dopamine (DA) is closely related to the functional structure of blood vessels. On day 30, the total content of DA in H4000-CD25/dCas9-sdTEVGs was significantly higher than that in the control group and close to that in normal rat blood vessels (Figure 6(h)). Similar results were observed in tests of collagen content and endothelialization and smooth muscle reconstruction, which demonstrated the favorable vascular remodeling and matrix reconstruction in H4000-CD25/dCas9-sdTEVGs (Figure 6(g) and Supplementary Figure 5). The three-dimensional reconstruction of the vascular adventitia using immunofluorescence revealed the effect of nerve reconstruction on sdTEVGs 1 to 3 months after transplantation. The signals of cord-like nerve fibers could be traced in the adventitia over time, while the nerve gradually grew from the transplanted end to the middle of the sdTEVGs (Figure 6(i); Supplementary Video 1-4). These data showed that the regenerated nerve fibers contribute to vascular matrix remodeling and reduced the risk of vascular stenosis.

Immune cell infiltration of implants and their effect on patency have attracted attention. A previous study has demonstrated that the host cell response often determines the fate of the explanted vascular grafts [31]. From the point of view of material biology, the immune response induced by vascular implant transplantation provides a new perspective for the regulation of regeneration. Antigen-presenting cells and lymphocytes trigger the chronic immune rejection of vascular grafts and lead to transplantation failure [32, 33]. With respect to the host inflammatory response, this study found that after sdTEVG transplantation into rats, T cells, macrophages, and a small amount of Treg cells were recruited over time and existed mainly within the adventitia. We revealed the spatiotemporal regularity of host immune cell infiltration after vascular implantation, which laid the foundation for the regulation of immunity and regeneration in situ. However, most commercially available synthetic grafts mainly induce inflammatory reactions, are rarely used to achieve regeneration, and fail to integrate properly into the surrounding tissue [34–36]. Nerve regeneration can regulate vascular activity and homeostasis. This can play an important role in extracellular matrix and normal smooth muscle remodeling and induce neuroimmune homeostasis to promote long-term symbiosis between the implant and host [37, 38].

However, excessive inflammatory reactions and immune rejection hinder the neural reconstruction of engineered blood vessels [39]. An initial objective of the project was to identify the relationship among immune homeostasis, nerve reconstruction, and artificial blood vessel regeneration.

Treg cells have strong immunomodulatory abilities, and their safety and efficiency has have been demonstrated in some a clinical trials [40]. We selected the CRISPR/dCas9 technology to promote TET2 expression to maintain the low methylation levels in the gene encoding a key factor Foxp3 and stabilize Treg cells to control inflammation. Furthermore, we modified the plasmid delivery nanoparticles with a CD25 antibody to target the Treg cells, and the transfection efficiency substantially improved. After TET2 expression in Treg cells was improved by the transfection, we found that not only was Foxp3 expression stabilized but also that Treg cells were actively expressing CD44. The increased secretion of anti-inflammatory factors TGF-*β*, IL-35, and IL-10 and inhibition of T-cell proliferation validated the anti-inflammatory capabilities of Treg cells, which were further amplified after gene editing, and were consistent with a previous finding that showed that the lack of TET2 in myeloid cells could lead to increased IL-6 secretion and atherosclerosis risk [41, 42].

Excessive inflammation is responsible for poor peripheral nerve regeneration in tissues [43], and proinflammatory populations of macrophages significantly suppress peripheral nerve repair [44]. Macrophages exist in different phenotypes depending on their environment. During nerve regeneration, chemokine C-C motif ligand 2 (CCL2) mRNA expression increases in axotomized neurons and Schwann cells, which is the major chemokine for monocytes and acts on the macrophage receptor CCR2 [45–47]. At the initial stage, CCR2+ macrophages activate neuronal signal transducer and activator of transcription 3 (STAT3), which promotes myelination by acting on Schwann cells, leading to the clearance of axonal debris, and removing molecules that inhibit nerve regeneration [48]. However, the long-term presence of CCR2+ macrophages results in excessive inflammation and delays wounded tissue healing. We cocultured macrophages and activated Treg cells and found that compared with that in the control group, the number of CCR2+ macrophages decreased. Furthermore, the levels of nerve growth-related factors NGF and Netrin-1 in the macrophage supernatant were significantly increased after stimulation with the Treg cell supernatant, whereas the level of IL-6 secreted by the macrophages was significantly reduced. IL-6 has been identified to be very important for peripheral nerve regeneration [28, 29]. IL-6 was upregulated during Wallerian degeneration after nerve injury, and delivery of IL-6 into injury sites could activate the signal transducer and activator of transcription 3 (STAT3) pathway, and then the downstream genes related to axon regeneration, such as growth-associated protein 43 (GAP43), leading to peripheral nerve axon growth [49–51]. Here, we found that in the low level-inflammation group, the IL-6 content decreased but the nerve cells grew better. However, nerve growth was worse than that in the control group after adding the IL-6 antagonist into the medium, which indicated that the positive effect of CCR2- macrophages on nerve growth may not only be attributed to NGF or Netrin-1 proteins but also the appropriate concentrations of IL-6. Thus, we further treated the neuron cells with a gradient concentration of IL-6 in vitro and found that the optimum concentration was 50–100 ng mL-1. Regulating the proportion of CCR2 macrophage subsets by editing Treg cells may promote the local IL-6 level in this optimal range. Future research on nerve regeneration will further confirm the effect of IL-6.

During the reinnervation process of vascular grafts, Schwann cells play important roles as they not only grow to link adjacent endplates in the denervated tissues but also resemble the miniature endplate potentials of innervated tissues [52, 53]. Thus, we cocultured the rat primary DRG of neurons and Schwann cells. The expression of S100 in different groups suggested that the supernatant of macrophages cultured with Treg cells promoted the combination of Schwann cells on axons, indicating that this interaction will promote the formation of a link between the injury nerve fibers and endplates in vascular grafts in vivo. We found that active Treg cells can promote the colocalization of nerve axons and Schwann cells, indicating that axons extend with the support of Schwann cells. Axonal regeneration is inseparable from the support and repair of Schwann cells. These findings suggest a positive role in both axonal regeneration and Schwann cell protection.

Thus, the H4000-CD25 encapsulated dCas9 system was attached onto the adventitia of sdTEVGs using a layer-by-layer electrostatic self-assembly method, which is widely used for the preparation of nanoparticles on various substrates for a range of applications [54]. The targeting nanoparticle transfection system could be stably released for 30 days in vivo, providing sufficient time for inflammation control and nerve fiber growth. Compared with injection or oral administration, the implantation of a sustained-release system requires a smaller dose and avoids systemic side effects, including hepatotoxicity and infection. The reporter gene GFP showed that the dCas9 was effective in vivo and mainly expressed in Treg cells at day 15 in the media and adventitia. The TET2 expression of sdTEVGs in the H4000-CD25/dCas9 group was significantly higher than that in the other two groups, and more macrophages were transformed into the CCR2- phenotype. The effect became more pronounced with the increase in implantation time after 15 days.

This suggested that the delivery system functions normally in vivo and becomes stable for a certain period. Furthermore, a higher density of tubblin3 and PGP9.5-positive neural fibers existed in the adventitia of the sdTEVGs in H4000-CD25/dCas9 group, and the tracer experiment further confirmed these neural fibers came from the superior cervical ganglion located next to the common carotid artery. In pathological conditions, smooth muscles without nerve connection exhibit pathological behaviors, such as abnormal proliferation and migration, and will not produce normal extracellular matrix or secrete extracellular matrix-degrading enzymes, such as matrix metalloproteinases, leading to conditions, such as vascular wall thickening, sclerosis, and aneurysm. The sustained-release system inhibited excessive inflammation and promoted nerve regeneration in situ. SdTEVGs innervated by neural fibers showed good patency and complete, regular morphological features. Nerve regeneration plays an important role in vascular extracellular matrix remodeling and regulation of vascular homeostasis. There is a harmonious interaction among the sustained-release system of scaffold materials, the improvement of local immune homeostasis, nerve remodeling, and good implant morphology and function. It is effective to promote the regeneration of engineered blood vessels from the point of view of immune regulation.

## 3. Conclusion

In summary, we established a gene editing system for the targeted delivery of Treg cells, which modulated the inflammatory environment of sdTEVGs for nerve regeneration by activating Treg cells and led to the phenotypic transformation of macrophages. Nerve-regulated sdTEVGs showed good extracellular matrix remodeling and patency. This type of advanced delivery system, with immunomodulatory function and neural recognition, contributes to the integration and functionalization of multiple grafts by the host.

## 4. Materials and Methods

### 4.1. Materials

Antibodies against CD68 (TA318150) were purchased from Origene Technologies. CD3-PE (550353), CD25-PE (554866), anti-CD3 (554829), anti-CD28 (554992), and anti-CD4-APC (550057) antibodies were purchased from BD. CD44-647 (203908) was obtained from Biolegend, anti-GAPDH antibody (gb11002) from Servicebio, and tocilizumab (m6223) from Abmole. Anti-PGP9.5 (ab8189), anti-Foxp3 (ab22510), and anti-CCR2 (ab203128) were purchased from Abcam. The antitubulin antibody (bs-4512r) was obtained from Bioss. The CRISPR/cas9 activation vector was purchased from Beijing Syngentech Co., and the nanotransfection reagent (H4000) from Engreen. The anti-MAG antibody (p100328) was purchased from Kleanab, secondary anti-mouse488 antibody (a11001) from Life Technology, and SC-1 (hy-10579) from MCE.

### 4.2. Animals

Animal procedures were reviewed and approved (the approved number: SCXK-PLA-20120011) by the animal ethics committee of Army Medical University. In total, 216 6-week-old male specific pathogen-free Sprague-Dawley (SD) rats (250–300 g) were provided by the animal house of Army Medical University. We used anesthesia with isoflurane (RWD Life Science, Shenzhen, China) to minimize pain during the organ extraction and sdTEVG implantation.

### 4.3. Experimental Design

The aim of this study was to promote the nerve regeneration of sdTEVGs. The effectiveness of the CRISPR/dCas9 nanodelivery system on nerve regeneration on sdTEVGs was investigated via in vitro and in vivo experiments. The in vitro experiments that evaluated the transfection of a CRISPR/dCas9 nanodelivery system on Treg cells and nerve fibers included flow cytometry, ELISA, RT-qPCR, and immunofluorescence. Rat primary DRG and peritoneal macrophages were used. The in vivo studies were conducted in 250 g female SD rats transplanted with sdTEVGs, and then nerve distribution, infiltration of macrophages and Treg cells, and the characteristics of sdTEVGs were measured. CRISPR/dCas9 nanodelivery system characteristics were investigated using scanning electron microscopy (SEM); zeta potential and stability were investigated in vitro and in vivo. The CRISPR/dCas9 nanodelivery system modified on sdTEVGs was not blinded. Investigators were blinded to sdTEVG transplantation in rats, macrophage phenotypes, Treg cell transfection, and characteristics of blood vessels. Sample processing and statistical analysis were performed concurrently on all groups using identical conditions. For all experiments, sample sizes, numbers of replicates, and statistical tests are indicated in the figure legends to ensure statistically significant differences.

### 4.4. Extraction and Culture of Treg Cells

The spleens extracted from SD rats were ground and sieved through a 40-mesh sieve. Cell suspensions were washed with PBS, and red blood cells were removed with lysate (Biosharp, BL503A). After resuspending the cells in RPMI (Solarbio), they were incubated with CD4-APC and CD25-PE, and the double-positive cell groups were screened using flow cytometry. Finally, cells were cultured in RPMI medium containing IL-2 (novo protein, final concentration of 30 ng mL-1) and 10% FBS and then seeded in a 12-well plate covered with CD3 (BD) and CD28 (BD).

### 4.5. dCas9 Plasmid Design

A 200 bp sequence, which was upstream of the initiation codon, was selected as the dCas9-sgRNA binding sequence to enhance TET2 expression. The sgRNA sequence was designed as follows: 5′-CCGCCCCGAGGCCGGCCGCCG-3′. The transfection vector was constructed and synthesized by Beijing SyngenTech Co. as follows: pZdonor_U6-sgRNA-EF1*α*-dSpCas9-NLS-VP64-2A-EGFP-2A-Puro.

### 4.6. Manufacturing of H4000-CD25PE and H4000-Cy3 Nanoparticles

The Entranster-H4000 (H4000, Engreen) was diluted from 20 *μ*L to 1 mL. Hydrodynamic dimension and zeta potential were measured. CD25-PE (100 *μ*L, 0.2 mg mL-1) was mixed with EDC (300 *μ*L, 1 mg mL-1) in MES (pH 5.5), and then, H4000 (100 *μ*L) was added overnight in the dark. Finally, H4000-CD25PE was purified using a dialysis bag with a molecular weight of 100 kD and enriched in 100 *μ*L of water. The hydrodynamic force and zeta potential of the product were measured again to check for coupling effect, and 20 *μ*L was used to perform the fluorescence spectrometer test. Activated Cy3-NHS (10 *μ*L, 5 mg mL-1), H4000 (200 *μ*L), and NaHCO3 (200 *μ*L, 0.1 M, pH 7.8) were homogeneously mixed and reacted overnight in the dark at 4°C. Subsequently, the reacted solution was dialyzed (molecular weight: 3500 D) in deionized water for 24 h, and the H4000-cy3 nanoparticles were obtained. Furthermore, H4000 and purified H4000-CY3 were diluted to 0.5 mg mL-1 with deionized water, and the hydration particle size and surface potential of each sample were tested thrice using a laser particle size analyzer (Malvern, zen3690).

### 4.7. dCas9 Plasmid Packaging in Entranster-H4000

The dCas9 plasmid and vector Entranster-H4000/H4000-CD25PE were diluted with serum-free RPMI to a working concentration of 32 *μ*g mL-1 and 80 *μ*g mL-1.

To prepare the transfection complex, the Entranster-H4000 was mixed with the plasmid at a volume ratio of 2 : 5 and kept for 15 min at 18–25°c. The following groups of transfected vectors and plasmids were mixed using the same methods: H4000-CD25PE, H4000/dCas9, and H4000-CD25PE/dCas9.

### 4.8. Transfection and Activation Rates

In total, 1 × 10^4^ Treg cells were cultured in a 96-well plate with normal medium (50 *μ*L) in each well for 24 h before transfection. Then, transfected vectors (20 *μ*L) from each group were added into the wells and incubated for 15 min at 25°C. After transfection for 6 h, the medium was replaced with a medium without transfection reagents. Two days later, these were purified using PURO antibiotics and showed green fluorescence (GFP). RT-PCR was also performed to detect TET2 expression in Treg cells to confirm the success of transfection at the mRNA level. Furthermore, Treg cells were incubated with a primary antibody against GFP (Servicebio) followed by secondary antibody (568 antirabbit) incubation, primary antibody against Foxp3 (Abcam), and secondary antibody (568 antimouse). Transfection efficiency was measured using flow cytometry (C6, BD).

To detect the transfection efficacy of H4000-CD25PE, Treg cells were mixed with T cells at a ratio of 1 : 1 and transfected using this method. Two days later, the cells were incubated with CD25PE, CD44-APC (BD), primary rabbit antibody against GFP (bs-0844r, BIOSs), and secondary antibody (568 antirabbit). The affinity and activation rates of Treg cells were measured using flow cytometry (C6, BD).

### 4.9. Real-Time qPCR

TET2 mRNA expression in Treg cells was compared before and after transfection. Briefly, total RNA was extracted using the Trizol reagent (Life), and cDNA was synthesized. The cDNA of different samples was amplified using StepOnePlus, and qPCR was performed using the SYBR Green qPCR Mix (Tiangen Biology) according to the manufacturer's instructions. The primer sequences used are shown in Supplementary Table 1.

### 4.10. Protein Expression

Treg cells were lysed using RIPA lysis buffer before and after transfection. Supernatant was obtained after centrifugation at 700 × g TET2 and cytokines, including IL-6, TGF-*β*, IL10, and IL35, were detected using an ELISA kit (Shanghai Xin Yu Biotech) following the manufacturer's instructions.

### 4.11. Mixed Lymphocyte Reaction Experiment

T cells (CD3-PE positive) and Treg cells (CD4-APC and CD25-PE double-positive) were obtained from the spleen, sorted using flow cytometry, and then cultured in RPMI (Solarbio) medium containing 30 ng mL-1 IL2 (Novoprotein Scientific) and 10% FBS. Cells in the control groups or those transfected with H4000-CD25PE/dCas9 were used at a 1 : 9 ratio to establish a mixed population of T cells and Treg cells. Finally, cells were, respectively, incubated with CD3PE and Foxp3-647 after culture for 1 day and 1 week. The proportion of T cells and Treg cells was compared using flow cytometry to determine the immunosuppressive effect of Treg cells.

### 4.12. Treatment of Macrophages with the Supernatant of Treg Cells

Peritoneal macrophages were obtained and cultured as previously described [55]. The supernatant of Treg cells transfected with H4000-CD25PE and H4000-CD25PE/dCas9 (rTreg and aTreg) was extracted, mixed with RPMI at the ratio of 1 : 1, and used to treat the macrophages for 2 days. Later, macrophage culture medium was changed to fresh RPMI for another 2 days' treat, and the supernatant of macrophages in each group was then extracted and preserved at -20°C for subsequent experiments. The phenotypes of macrophages in three groups were, respectively, determined using immunofluorescence staining. Macrophages were incubated with mouse anti-CD68 (OriGene Technologies, 1 : 500) and rabbit anti-CCR2 (Servicebio, 1 : 500) antibodies overnight at 4°C and washed with PBS (5 min × 3 times) before adding the secondary antibodies (Alexa Fluor 488 antimouse and 568 antirabbit). The cells were imaged using a confocal imaging system (LSM780).

### 4.13. Treatment of Neurons with the Supernatant of Macrophages

After sterilization with alcohol immersion, the back skins of 1-day-old SD rats were cut longitudinally along the spine. The DRG on both sides was taken out, and the fascia was removed as much as possible. These were cut into 1 mm^3^ pieces and transferred into a 0.25% trypsin digestion solution for 20 min at 37°C. Dulbecco's modified Eagle medium (DMEM) with 10% FBS was added to stop the digestion, and the supernatant was discarded after centrifugation at 150 × g for 10 min. The neuron cells were cultured on polylysine-coated slides in a complete medium (98%neuron basic medium − A + 1.5%B27 supplement (Gibco) + 0.5%penicillin and streptomycin). After 1 day, the neurons were divided into four groups, respectively, treated by complete neuron medium and the three groups of macrophage supernatants in a 1 : 1 ratio, and fourth group was treated as the third group supplemented with tocilizumab (100 *μ*g mL-1, IL6R antagonist, Abmole, M6223). After two days of culture, immunofluorescence staining for PGP9.5 (Abcam) was performed. The growth of axons was detected using fluorescent imaging.

### 4.14. Schwann Cell Culture

After sterilization with alcohol immersion, the sciatic nerves were removed from neonatal SD rats (1–3 days old), and the neurilemma was carefully removed. Nerves were digested with type IV collagenase (1.6 mg mL-1) for 10 min at 37°C, and then, trypsin was added at a 1 : 1 ratio to continue the digestion for 5 min. After centrifugation at 150 × g for 5 min, the cell pellet was suspended in DMEM medium with 10% FBS and placed in a cell plate for culture. Two days later, trypsin was added for digestion until most of the Schwann cells and a few fibroblasts were digested and then replaced twice in the cell plate for culture according to the differential detachment protocol for purification [56]. After repeated cultivation, the Schwann cells were purified.

### 4.15. Coculture of Neurons and Schwann Cells

After culture, purification, and identification, Schwann cells were digested with 0.125% trypsin for 5 min and placed into DMEM medium containing VC (250 *μ*M) and FBS (100 mL L-1). Then, Schwann cells were cocultured with primary neurons cells at a ratio of 10 : 1 for 3 to 5 days to promote their combination. Macrophage supernatants from above three groups (had mixed with Treg cells supernatants) were added into the medium at the ratio of 1 : 1 for another 2-day culture. Finally, Schwann cell growth on nerve axons was detected using immunofluorescence staining with S100 (KLEANAB) and PGP9.5 (Abcam).

### 4.16. Preparation of sdTEVGs

Under sterile conditions, the carotid arteries of SD rats were harvested and decellularized with 0.05% trypsin (Gibco) at 37°C for 30 min and washed thrice with PBS for 5 min. Subsequently, acellular blood vessels were soaked in h4000-cd25pe, h4000/dCas9, and h4000-cd25pe/dCas9 for 10 min, washed with PBS for 2 min, and again soaked in a soluble collagen PBS solution (1 g L-1) for 10 min. SdTEVGs were obtained after repeating the incubation and elution thrice.

### 4.17. Structure of sdTEVGs Modified with Transfected Vectors

Five groups, namely, three transfected nanoparticles groups (H4000, H4000-CD25, and H4000-CD25/dCas9) and two blood vessel groups (acellular blood vessels or incubated with H4000-CD25/dCas9), were investigated. Before SEM observation, a drop of transfected nanoparticles was added into the mica sheet and dried for the first three groups. The blood vessels in the last two groups needed to be fixed with glutaraldehyde overnight and then gradually dehydrated by alcohol and tert-butyl alcohol. All samples were fixed on the carrier, sprayed with gold, and photographed using SEM (Zeiss).

### 4.18. Surface Zeta Potential Detection

The porcine thoracic aorta was cut into square slices of 0.8 cm × 0.8 cm. A conductive adhesive was used to stick to the glass substrate. Samples were activated by adding EDC-NHS (1 mg mL-1) and incubating for 2 h. Afterward, blood vessels were transferred to 1% hyaluronic acid solution and incubated at 37°C for 2 h. After gently soaking with deionized water for 5 min, the blood vessels were transferred to H4000-dCas9 (0.2 mg mL-1) solution for 2 h. After gently washing with deionized water, the procedure was repeated 3–5 times. The final blood vessels were tested with A surface potentiometer (ZetaCAD).

### 4.19. Sustained Release of H4000 on sdTEVGs In Vitro and In Vivo

SdTEVGs were modified with H4000-Cy3 using the above method and then placed in a 1.5% agarose physiological saline gel. Finally, the residual fluorescence of Cy3 was photographed and compared at 1, 3, 7, 15, and 30 days. Furthermore, H4000-DIR was used to modify the sdTEVGs and then transplanted into rats. At 3, 7, 15, and 30 days after transplantation, the skin hair around the surface layer of the carotid artery was removed, and rats were then analyzed using in vivo imaging system Spectrum (PerkinElmer) at 740 nm and imaged using the Living Image software (version 4.4).

### 4.20. Plasmid Release

The following PCR primers were designed for the GFP fragment on the dCas9 plasmid using Premier 5.0: JCs: 5′-GAAGAACGGCATCAAGGTG-3′ and JCa: 5′-ACTGGGTGCTCAGGTAGTGG-3′, and H4000-CD25PE/dCas9 modified sdTEVGs were placed in 1.5% agarose physiological saline gel; gel (2 *μ*L) with released plasmid was taken out at 1, 3, 7, 15, and 30 days and mixed into a 10 *μ*L PCR system for amplification. The brightness of electrophoretic bands was analyzed using ImageJ to calculate the plasmid sustained-release rate.

#### 4.20.1. SdTEVG Transplantation

All sdTEVGs (length: 1 cm, diameter: 1 mm) in the H4000-CD25PE, H4000/dCas9, and H4000-CD25PE/dCas9 groups were transplanted into the carotid artery of 6–8-week allogenic SD rats, which were injected intraperitoneally with heparin sodium (1,000 U kg-1, Aladdin) for 4 days, and maintained in the absence of a specific pathogen.

### 4.21. Ultrasound and CT Detection of sdTEVGs in SD Rats

Thirty days after transplantation, the SD rats in the three groups (5 samples in each group) were anesthetized and evenly smeared with ultrasonic gel in the neck. The blood flow and waveform of the carotid artery were observed using Doppler ultrasound, and the flow velocity was statistically compared. After injecting heparin (1 mL) through the tail vein, iohexol (300 mg mL-1, Tianheng Pharmaceutical) was injected into the left ventricle at 1.5–2 mL kg-1. The SD rats were scanned using the small animal CT scanner (Skyscan 176, Bruker micro CT), and CT images were obtained to characterize the patency of the sdTEVGs.

### 4.22. Protein Detection in sdTEVGs

SdTEVGs were cut into 2 mm pieces and soaked in RIPA lysate (Beyotime) at a proportion of 10% (*w*/*v*), sonicated on ice thrice for 30 s with an Ultrasonic Crasher Noise Isolating Chamber (SCIENTZ, Ningbo Science Biotechnol Co.), and centrifuged at 1,200 × g to obtain the supernatant. An ELISA Kit (Xin Yu Biotech) was used to detect IL-6 and DA in sdTEVGs 1, 3, 7, and 15 days after transplantation. A hydroxyproline (Hyp) kit (Xin Yu Biotech) was used to detect collagen in sdTEVGs. To detect TET2 expression, the supernatant (60 *μ*g) was heated at 95°C and transferred for electrophoresis and western blotting. Blots were probed with anti-TET2 (Abclone, 1 : 500) and anti-GAPDH antibodies (Bioworld).

### 4.23. Virus Injection into the Stellate Ganglion (SG)

Two months after transplantation, SD rats were anesthetized, and the SG nearby sdTEVGs were carefully separated to avoid damaging the peripheral nerves. A 2.5 *μ*L titer 1.06*e* + 13 AAV virus AOV002 (Heyuan, sequence: paav-cag-mcs-egfp-3flag, serotype AAV2/9) was then injected into the SG using 3.5” glass capillaries (Drummond). Five days later, SG and sdTEVGs were extracted and fixed with 4% paraformaldehyde. The SG frozen sections and adventitia of sdTEVGs were imaged using a confocal imaging system.

### 4.24. H&E and Immunofluorescence Staining

SdTEVGs in three groups (H4000-CD25PE, H4000/dCas9, and H4000-CD25PE/dCas9) were taken out 3, 7, and 15 days after implantation for immunofluorescence staining and at 3 months for H&E staining. In addition, sdTEVGs without transfection in the control group were taken out at 30 days for immunofluorescence staining. SdTEVGs were embedded in paraffin, sectioned (5 *μ*m thick), dewaxed in xylene, rehydrated in an alcohol gradient, and stained by H&E. Sections in each group were incubated with antirabbit GFP (Servicebio, 1 : 500), antimouse Foxp3 (Abcam, 1 : 500), and antimouse CD68 antibody (OriGene Technologies, 1 : 500), followed by detection with an Alexa Fluor 488 secondary antibody (Thermo Fisher, 1 : 1,000), antirabbit CCR2 antibody (Abcam,1 : 500), Alexa Fluor 568 secondary antibody (Thermo Fisher, 1 : 1,000), and 4′,6-diamidino-2-phenylindole (DAPI, Invitrogen) to stain the cell nuclei at room temperature for 7 min. SdTEVGs in the control group were incubated with antirabbit CD3 (Servicebio, 1 : 500), antimouse Foxp3 (Abcam, 1 : 500), antimouse CD68 antibody, and antirabbit CCR2 antibody followed by the indicated secondary antibodies. Confocal photography was performed, so that the temporal and spatial law of immune cells can infiltrate the transplanted blood vessels with or without transfection.

### 4.25. Three-Dimensional Reconstruction

The sdTEVGs in the H4000-CD25PE/dCas9 group were taken out at 1, 2, and 3 months. After washing and fixation, the adventitia was torn and stretched for prepared slices. Subsequently, slices were incubated with antimouse PGP9.5 (Abcam, 1 : 500) followed by Alexa Fluor 488 secondary antibody and antirabbit TUBB3 (Bioss, 1 : 500) followed by an Alexa Fluor 568 secondary antibody (Thermo Fisher, 1 : 1,000). Confocal images were taken, and 3D Max 2020 software (Autodesk) was used to reconstruct the vascular nerve.

### 4.26. Statistical Analysis

Data were analyzed using the GraphPad Prism 7.0 software and are expressed as mean ± SEM. Unpaired Student's *t*-test was used to analysis single comparisons between two independent groups. One or two-way ANOVA followed by Tukey test analysis was used to analyze the variance (ANOVA) of multiple groups. *P* values of ^∗^*P* < 0.05, ^∗∗^*P* < 0.01, and ^∗∗∗^*P* < 0.001 were considered statistically significant.

## Data Availability

All data supporting the findings of this study are available within the paper and its supplementary information.

## Supplementary Materials

Supplementary Materials Supplementary Figure 1: TET2 protein levels in Treg cells stimulated with the inhibitor SC1 and activator VITC, as determined using ELISA. Supplementary Figure 2: transfection efficiency of the H4000-CD25/dCas9 delivery system in vitro. Supplementary Figure 3: IL-6 stimulation of the dorsal root ganglion. Supplementary Figure 4: Sustained release detection of H4000-Cy3 modified on sdTEVG adventitia in a 1.5% agarose physiological saline gel. Supplementary Figure 5: evaluation of ECs and SMCs regeneration and remodeling in explanted grafts at 1 month after implantation. Supplementary Table 1: primer sequences used for real-time quantitative polymerase chain reaction. Supplementary Video 1: three-dimensional reconstruction of immunofluorescence-stained adventitia of normal rat common carotid artery. Supplementary Video 2: three-dimensional reconstruction of immunofluorescence-stained adventitia after 1 month of sdTEVG transplantation. Supplementary Video 3: three-dimensional reconstruction of immunofluorescence-stained adventitia after 2 months of sdTEVG transplantation. Supplementary Video 4: three-dimensional reconstruction of immunofluorescence-stained adventitia after 3 months of sdTEVG transplantation.

## References

[1] Niklason L. E., Lawson J. H. (2020). Bioengineered human blood vessels. *Science*.

[2] Mou X., Zhang H., Qiu H. (2022). Mussel-inspired and bioclickable peptide engineered surface to combat thrombosis and infection. *Research*.

[3] Luo J., Qin L., Zhao L., Gui L., Qyang Y. (2020). Tissue-engineered vascular grafts with advanced mechanical strength from human iPSCs. *Cell Stem Cell*.

[4] Ruhoff A. M., Hong J. K., Gao L., Singh J., Waterhouse A. (2021). Biomaterial wettability affects fibrin clot structure and fibrinolysis. *Advanced Healthcare Materials*.

[5] Xin Z. A., Qiang G. B., Dan Y. A. (2022). 3D-bioprinted vascular scaffold with tunable mechanical properties for simulating and promoting neo-vascularization. *Smart Materials in Medicine*.

[6] Zhang Y., Liu Y., Jiang Z., Wang J., Zhao H. (2021). Poly(glyceryl sebacate)/silk fibroin small-diameter artificial blood vessels with good elasticity and compliance. *Smart Materials in Medicine*.

[7] Lee P. P., Cerchiari A., Desai T. A. (2014). Nitinol-based nanotubular coatings for the modulation of human vascular cell function. *Nano Letters*.

[8] Matsuyama A., Takatori S., Sone Y. (2017). Effect of nerve growth factor on innervation of perivascular nerves in neovasculatures of mouse cornea. *Biological & Pharmaceutical Bulletin*.

[9] Sun Y., Yang Z., Zheng B. (2017). A novel regulatory mechanism of smooth muscle *α*-actin expression by NRG-1/circACTA2/miR-548f-5p axis. *Circulation Research*.

[10] Wen Z., Wei Y., Li L. (2010). The promotion of endothelial progenitor cells recruitment by nerve growth factors in tissue-engineered blood vessels. *Biomaterials*.

[11] Wen Z., Wen C., Wu Y. (2012). The use of BDNF to enhance the patency rate of small-diameter tissue- engineered blood vessels through stem cell homing mechanisms. *Biomaterials*.

[12] Li Y., Wan S., Liu G. (2017). Netrin-1 promotes inflammation resolution to achieve endothelialization of small-diameter tissue engineering blood vessels by improving endothelial progenitor cells function in situ. *Advanced Science*.

[13] Roh J. D., Sawh-Martinez R., Brennan M. P. (2010). Tissue-engineered vascular grafts transform into mature blood vessels via an inflammation-mediated process of vascular remodeling. *Proceedings of the National Academy of Sciences of the United States of America*.

[14] Fontenot J. D., Gavin M. A., Rudensky A. Y. (2003). Pillars article: Foxp3 programs the development and function of CD4+CD25+ regulatory T cells. *Nature immunology*.

[15] Zhang H., Li Y., de Carvalho-Barbosa M. (2016). Dorsal Root Ganglion Infiltration by Macrophages Contributes to Paclitaxel Chemotherapy-Induced Peripheral Neuropathy. *Journal of Pain*.

[16] Ali N., Zirak B., Rodriguez R. S. (2017). Regulatory T cells in skin facilitate epithelial stem cell differentiation. *Cell*.

[17] Mata R., Yao Y., Cao W., Ding J., Gao C. (2021). The dynamic inflammatory tissue microenvironment: signality and disease therapy by biomaterials. *Research*.

[18] Ruili Y., Cunye Q., Yu Z. (2015). Hydrogen Sulfide Promotes Tet1- and Tet2-Mediated *Foxp3* Demethylation to Drive Regulatory T Cell Differentiation and Maintain Immune Homeostasis. *Immunity*.

[19] Braza M. S., Mmt Van Leent M., Lameijer B. L. (2018). Inhibiting inflammation with myeloid cell-specific nanobiologics promotes organ transplant acceptance. *Immunity*.

[20] (2020). Applicability, safety, and biological activity of regulatory T cell therapy in liver transplantation. *American Journal of Transplantation*.

[21] Yue X., Samaniego-Castruita D., González-Avalos E., Li X., Barwick B. G., Rao A. (2021). Whole-genome analysis of TET dioxygenase function in regulatory T cells. *EMBO REPORTS*.

[22] Ohkura N., Hamaguchi M., Morikawa H. (2012). T cell receptor stimulation-induced epigenetic changes and Foxp3 expression are independent and complementary events required for Treg cell development. *Immunity*.

[23] Qian Y., Han Q., Zhao X., Li H., Yuan W. E., Fan C. (2019). Asymmetrical 3D nanoceria channel for severe neurological defect regeneration. *Iscience*.

[24] Ych A., Fw B., Jl A., Sg A. (2022). The increased ratio of Mg^2+^/Ca^2+^ from degrading magnesium alloys directs macrophage fate for functionalized growth of endothelial cells. *Smart Materials in Medicine*.

[25] Xue Y., He J., Xiao C. (2018). The mouse autonomic nervous system modulates inflammation and epithelial renewal after corneal abrasion through the activation of distinct local macrophages. *Mucosal Immunology*.

[26] Siebert H., Sachse A., Kuziel W. A., Maeda N., Brück W. (2000). The chemokine receptor CCR2 is involved in macrophage recruitment to the injured peripheral nervous system. *Journal of Neuroimmunology*.

[27] Rosenberg A. F., Wolman M. A., Franzini-Armstrong C., Granato M. (2012). In vivo nerve-macrophage interactions following peripheral nerve injury. *Journal of Neuroscience the Official Journal of the Society for Neuroscience*.

[28] Ann C. A., Yves S., Gael H. (2017). Low-dose pulsatile interleukin-6 as a treatment option for diabetic peripheral neuropathy. *Frontiers in Endocrinology*.

[29] Shen Y., Guo Y., Mikus P., Sulniute R., Li J. (2012). Plasminogen is a key proinflammatory regulator that accelerates the healing of acute and diabetic wounds. *Blood*.

[30] Carr M. J., Toma J. S., Johnston A. P. (2019). Mesenchymal precursor cells in adult nerves contribute to mammalian tissue repair and regeneration. *Cell Stem Cell*.

[31] Kirkton R. D., Prichard H. L., Santiago-Maysonet M., Niklason L. E., Lawson J. H., Dahl S. L. M. (2018). Susceptibility of ePTFE vascular grafts and bioengineered human acellular vessels to infection. *Journal of Surgical Research: Clinical and Laboratory Investigation*.

[32] Ricco J. B., Gargiulo M. (2017). Commentary on "late dacron patch inflammatory reaction after carotid endarterectomy". *European Journal of Vascular & Endovascular Surgery the Official Journal of the European Society for Vascular Surgery*.

[33] Roy-Chaudhury P., Kelly B. S., Miller M. A., Reaves A., Heffelfinger S. C. (2001). Venous neointimal hyperplasia in polytetrafluoroethylene dialysis grafts. *Kidney International*.

[34] Stadtmauer E. A., Fraietta J. A., Davis M. M., Cohen A. D., June C. H. (2020). CRISPR-engineered T cells in patients with refractory cancer. *Science*.

[35] José J., Fuster S., MacLauchlan A. M., Zuriaga N. M., Polackal C. A., Ostriker (2017). Clonal Hematopoiesis Associated with TET2 Deficiency Accelerates Atherosclerosis Development in Mice. *Science*.

[36] Jaiswal S., Natarajan P., Silver A. J. (2017). Clonal hematopoiesis and risk of atherosclerotic cardiovascular disease. *The New England Journal of Medicine*.

[37] Dong X., Liu S., Yang Y., Gao S., Kong D. (2021). Aligned microfiber-induced macrophage polarization to guide Schwann-cell-enabled peripheral nerve regeneration. *Biomaterials*.

[38] Robert B., Alexander S., Michael R. (2018). Inflammaging impairs peripheral nerve maintenance and regeneration. *Aging Cell*.

[39] Panee J. (2012). Monocyte chemoattractant protein 1 (MCP-1) in obesity and diabetes. *Cytokine*.

[40] Du L., He H., Xiao Z. (2022). GSH-responsive metal-organic framework for intratumoral release of NO and IDO inhibitor to enhance antitumor immunotherapy. *Small*.

[41] Liu T., Soong L., Liu G., Nig R. K., Chopra A. K. (2009). CD44 expression positively correlates with Foxp3 expression and suppressive function of CD4+ Treg cells. *Biology Direct*.

[42] Lin Y., Wu J., Weihuai G. (2018). Exosome–liposome hybrid nanoparticles deliver CRISPR/Cas9 system in MSCs. *Advanced Science*.

[43] Gadient R. A., Otten U. H. (1997). Interleukin-6 (IL-6)--a molecule with both beneficial and destructive potentials. *Progress in Neurobiology*.

[44] Wang T., Wei J. J., Sabatini D. M., Lander E. S. (2014). Genetic screens in human cells using the CRISPR-Cas9 system. *Science*.

[45] Chen Y., Wang L., Kang Q. (2017). Heat shock protein A12B protects vascular endothelial cells against sepsis-induced acute lung injury in mice. *Cellular Physiology and Biochemistry*.

[46] Raghu H., Lepus C. M., Wang Q. (2017). CCL2/CCR2, but not CCL5/CCR5, mediates monocyte recruitment, inflammation and cartilage destruction in osteoarthritis. *Annals of the Rheumatic Diseases*.

[47] Love F. M., Son Y. J., Thompson W. J. (2003). Activity alters muscle reinnervation and terminal sprouting by reducing the number of Schwann cell pathways that grow to link synaptic sites. *Developmental Neurobiology*.

[48] Lindborg J. A., Mack M., Zigmond R. E. (2017). Neutrophils are critical for myelin removal in a peripheral nerve injury model of Wallerian degeneration. *Journal of Neuroscience the Official Journal of the Society for Neuroscience*.

[49] Tyrrell D. J., Goldstein D. R. (2021). Ageing and Atherosclerosis: Vascular Intrinsic and Extrinsic Factors and Potential Role of IL-6. *Nature Reviews Cardiology*.

[50] Land W. G. (2005). The role of postischemic reperfusion injury and other nonantigen-dependent inflammatory pathways in transplantation. *Transplantation*.

[51] Crupi A., Costa A., Tarnok A., Melzer S., Teodori L. (2005). Inflammation in tissue engineering: the Janus between engraftment and rejection. *European Journal of Immunology*.

[52] Carmeliet P., Tessier-Lavigne M. (2005). Common mechanisms of nerve and blood vessel wiring. *Nature*.

[53] Santosa S. M., Guo K., Yamakawa M. (2020). Simultaneous fluorescence imaging of distinct nerve and blood vessel patterns in dual Thy1-YFP and Flt1-DsRed transgenic mice. *Angiogenesis*.

[54] Yong H., Rawji K. S., Ghorbani S., Xue M., Yong V. W. (2019). The benefits of neuroinflammation for the repair of the injured central nervous system. *Cellular & Molecular Immunology*.

[55] Martinez-Rojas V. A., Salinas-Abarca A. B., Gomez-Viquez N. L., Granados-Soto V., Mercado F., Murbartian J. (2021). Interaction of NHE1 and TRPA1 Activity in DRG Neurons Isolated from Adult Rats and its Role in Inflammatory Nociception. *Neuroscience*.

[56] Wu W., Jin Y. Q., Kretlow J. D., Xu L., Duan H. C., Qi Z. L. (2009). Purification of Schwann cells from adult rats by differential detachment. *Biotechnology Letters*.

