# Supplementary material for: Programming of Regulatory T Cells In Situ for Nerve Regeneration and Long-Term Patency of Vascular Grafts
Source: Research (Wash D C). 2022 Jul 19;2022:9826426. doi: 10.34133/2022/9826426 (PMC9351587; doi:10.34133/2022/9826426)
Supplement: Supplementary Materials — Supplementary Figure 1: TET2 protein levels in Treg cells stimulated with the inhibitor SC1 and activator VITC, as determined using ELISA. Supplementary Figure 2: transfection efficiency of the H4000-CD25/dCas9 delivery system in vitro. Supplementary Figure 3: IL-6 stimulation of the dorsal root ganglion. Supplementary Figure 4: Sustained release detection of H4000-Cy3 modified on sdTEVG adventitia in a 1.5% agarose physiological saline gel. Supplementary Figure 5: evaluation of ECs and SMCs regeneration and remodeling in explanted grafts at 1 month after implantation. Supplementary Table 1: primer sequences used for real-time quantitative polymerase chain reaction. Supplementary Video 1: three-dimensional reconstruction of immunofluorescence-stained adventitia of normal rat common carotid artery. Supplementary Video 2: three-dimensional reconstruction of immunofluorescence-stained adventitia after 1 month of sdTEVG transplantation. Supplementary Video 3: three-dimensional reconstruction of immunofluorescence-stained adventitia after 2 months of sdTEVG transplantation. Supplementary Video 4: three-dimensional reconstruction of immunofluorescence-stained adventitia after 3 months of sdTEVG transplantation. [file 9826426.f1.zip › Supplementary Information.docx]

**Supporting Information**

**Programming of regulatory T cells in situ for nerve regeneration and long-term patency of vascular grafts**

Yanhong Wang^1†^, Fangchao Xue^1†^, Yanzhao Li^2†^,Lin Lin^1^, Yeqin Wang^1^, Shanlan Zhao^1^, Xingli Zhao^1^, Yong Liu^2^, Ju Tan^2^, Gang Li^2^, Haoran Xiao^1^, Juan Yan^1^, Hao Tian^1^, Min Liu^1^, Qiao Zhang^1^, Zhaojing Ba^1^, Lang He^1^, Wenyan Zhao^1^, Chuhong Zhu^2^ and Wen Zeng^1,3,4*^

^1^ Department of Cell Biology, Third Military Army Medical University, Chongqing 400038, China

^2^ Department of Anatomy, National and Regional Engineering Laboratory of Tissue Engineering, State and Local Joint Engineering Laboratory for Vascular Implants, Key Lab for Biomechanics and Tissue Engineering of Chongqing, Third Military Medical University, Chongqing 400038, China

^3^ State Key Laboratory of Trauma, Burn and Combined Injury, Chongqing, China

^4^ Departments of Neurology, Southwest Hospital, Third Military Medical University, Chongqing, China

†These authors contributed equally to this work.

***Corresponding Author**

Wen Zeng

Department of Cell Biology, National & Regional Engineering Laboratory of Tissue Engineering

Key Lab for Biomechanics of Chongqing, Third Military Medical University

Gao Tan Yan Street, Shaping Ba District, Chongqing 400038, China

Phone number: 13647638478

Email: [zengw0105@163.com](mailto:zengw0105@163.com)

Fax: 86-23 68771290


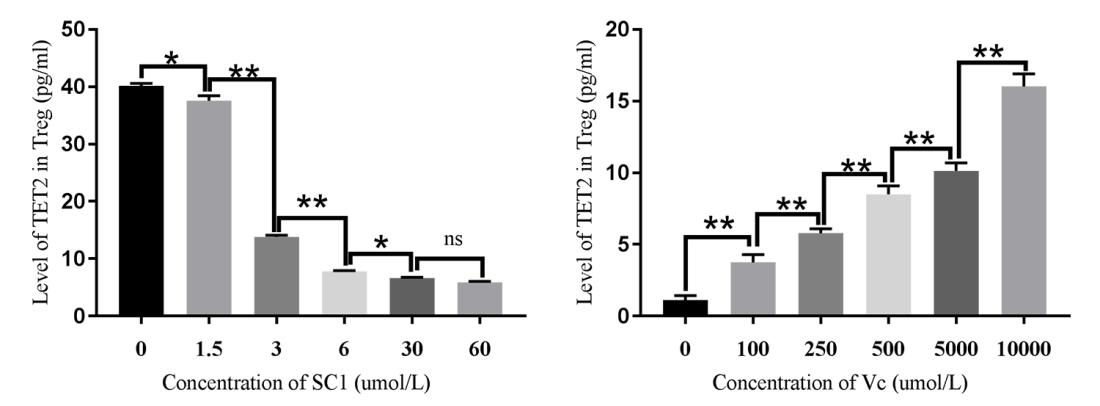


**Supplementary Figure 1.** TET2 protein levels in Treg cells stimulated with the inhibitor SC1 and activator VITC, as determined by ELISA. TET2, ten-eleven translocation-2; VITC, vitamin C; n = 6, one-way ANOVA. *P < 0.05, **P < 0.01, n.s., no significance. Data are presented as means ± SD


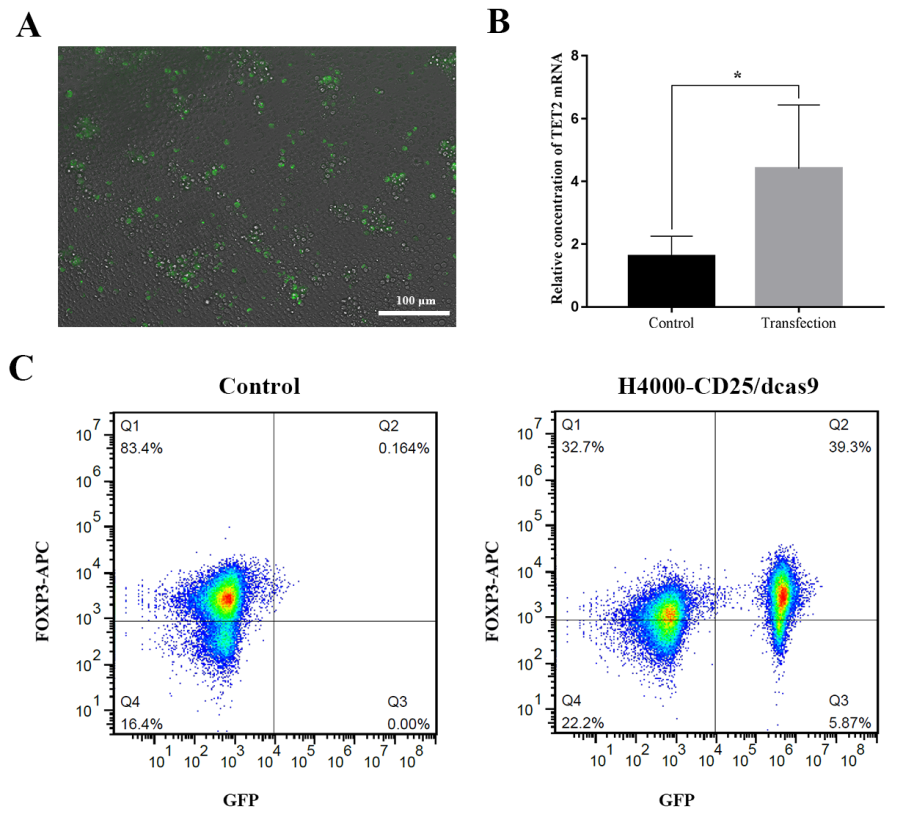


**Supplementary Figure 2**. Transfection efficiency of the H4000-CD25/dCas9 delivery system in vitro. A) Green fluorescent protein confocal image revealed the successful transfection of Treg cells. n = 6. B) qPCR-based detection of TET2 mRNA in Treg cells after transfection. n = 6, Student’s t-test. *P < 0.05. Data are presented as means ± SD. C) Analysis of the efficiency of Treg cells transfected with the H4000-CD25/dCas9 delivery system in vitro using flow cytometry. TET2, ten-eleven translocation-2; n = 6.


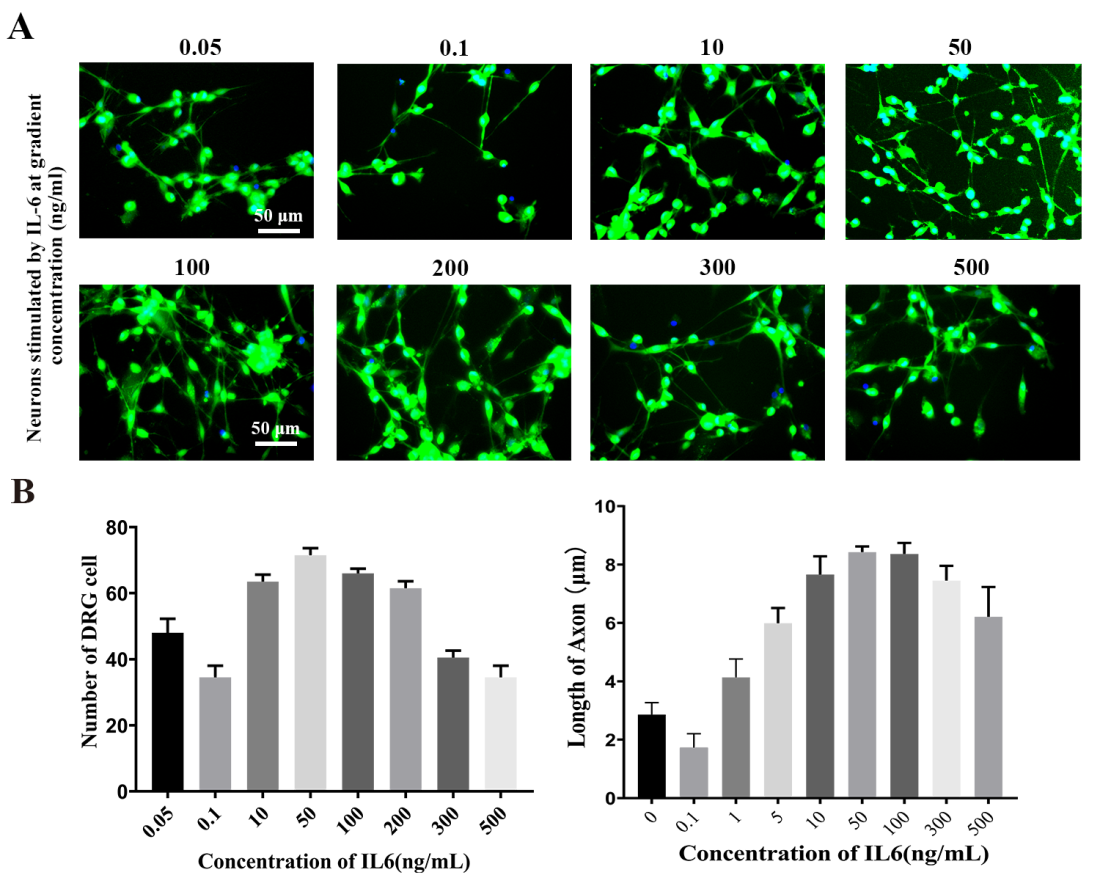


**Supplementary Figure 3**. IL-6 stimulation of the dorsal root ganglion. A) The rat dorsal root ganglion investigated using immunofluorescence under stimulation with different concentrations of IL-6; green, PGP9.5; blue. DAPI. B) Axonal length and number of surviving cells in the rat dorsal root ganglion after stimulation with different concentrations of IL-6. IL-6, interleukin 6; n = 6. Data are presented as means ± SD.


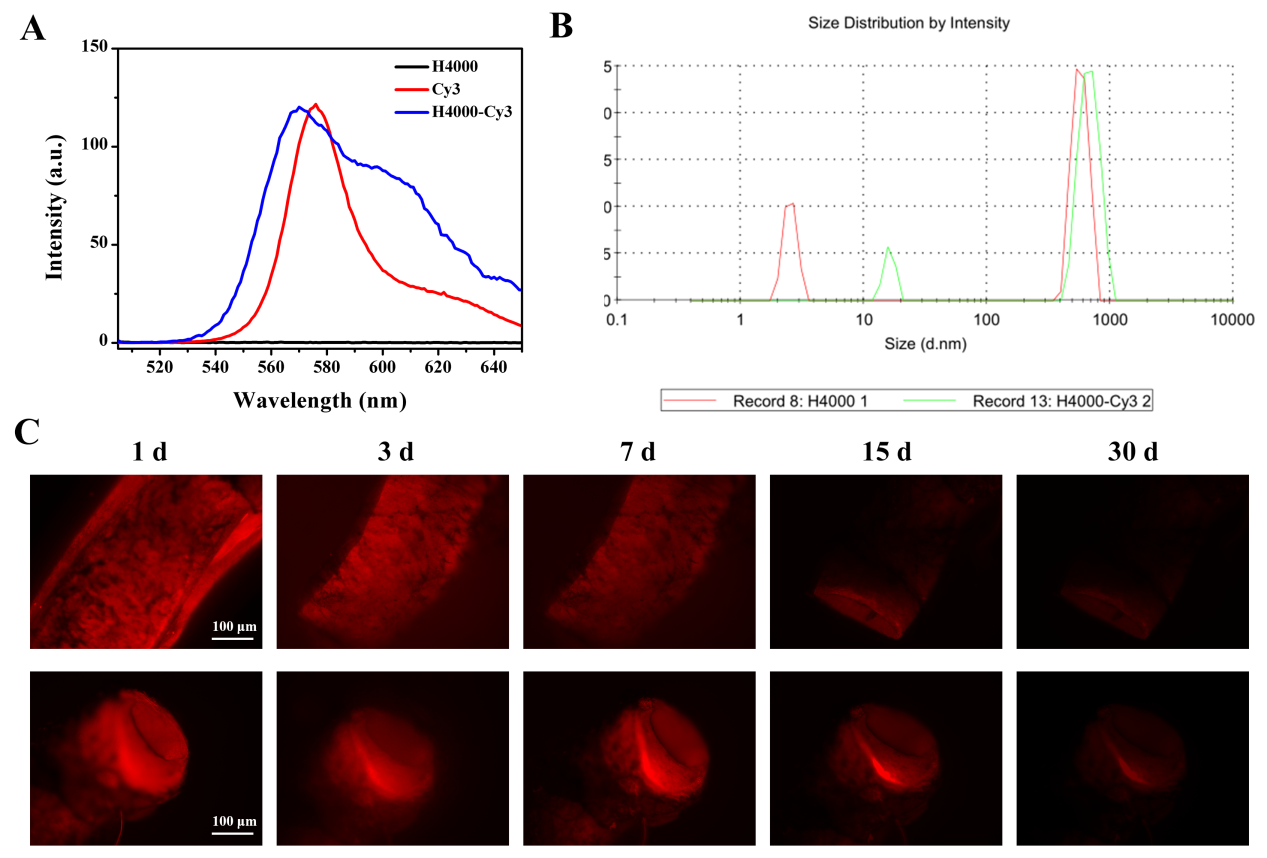


**Supplementary Figure 4**. Sustained release detection of H4000-Cy3 modified on sdTEVG adventitia in a 1.5% agarose physiological saline gel. A) H4000-Cy3 infrared spectrum analysis before and after the covalent binding of H4000 and Cy3. B) H4000-Cy3 particle size. C) The in vitro sustained-release time of engineered blood vessels modified with H4000-Cy3 detected using a fluorescence microscope; n = 6. Data are presented as means ± SD.


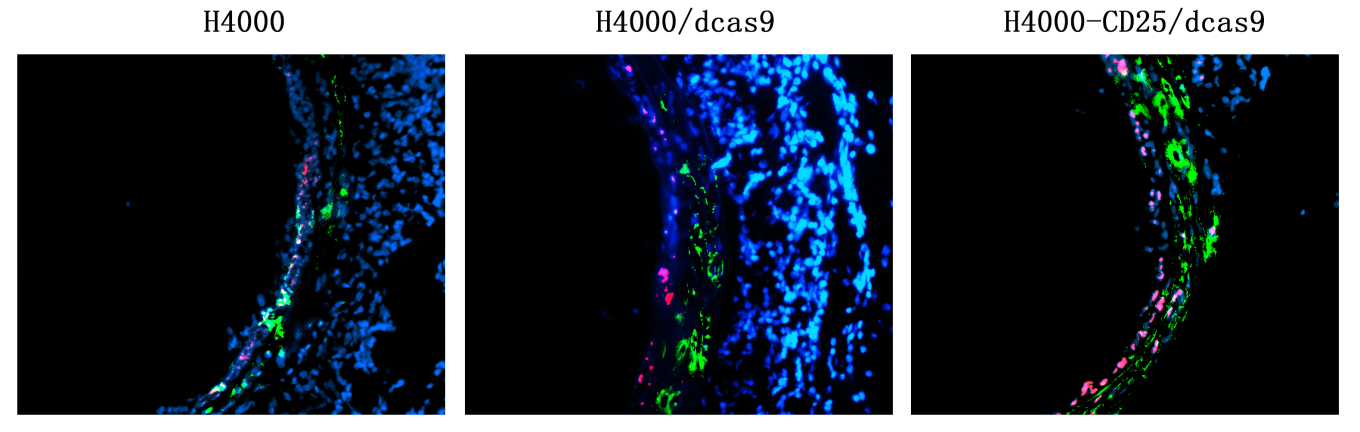


**Supplementary Figure 5**. Evaluation of ECs and SMCs regeneration and remodeling in explanted grafts at 1 month after implantation. Endothelialization and SMCs regeneration was analyzed by co-immunofluorescence staining of the explanted grafts using CD31 (red) and a-SMA (green) antibodies.

**Supplementary Table 1. Primer sequences used for real-time quantitative polymerase chain reaction**

| TET2 | S | 5-TCTGTGGAGGACATTGAGGTT -3 |
| --- | --- | --- |
|  | As | 5-ATTGGGCTTGTTTACTTTGGT -3 |
| GAPDH | S | 5- CGTGGAGTCTACTGGCGTCTT -3 |
|  | As | 5- ATTGCTGACAATCTTGAGGGAG -3 |

**Supplementary Video 1. Three-dimensional reconstruction of immunofluorescence-stained adventitia of normal rat common carotid artery.**

**Supplementary Video 2. Three-dimensional reconstruction of immunofluorescence-stained adventitia after 1 month of sdTEVG transplantation.**

**Supplementary Video 3. Three-dimensional reconstruction of immunofluorescence-stained adventitia after 2 months of sdTEVG transplantation.**

**Supplementary Video 4. Three-dimensional reconstruction of immunofluorescence-stained adventitia after 3 months of sdTEVG transplantation.**
